# Externally Triggered Activation of Nanostructure-Masked Cell-Penetrating Peptides

**DOI:** 10.3390/molecules30153205

**Published:** 2025-07-30

**Authors:** Gayong Shim

**Affiliations:** 1 School of Systems Biomedical Science, Soongsil University, Seoul 06978, Republic of Korea; shim@ssu.ac.kr; Tel.: +82-2-820-0451; 2 Integrative Institute of Basic Sciences, Soongsil University, Seoul 06978, Republic of Korea

**Keywords:** cell-penetrating peptide, stimuli-responsive delivery, cold atmospheric plasma, DNA masking, peptide activation, tumor spheroid

## Abstract

Cell-penetrating peptides offer a promising strategy for intracellular delivery; however, non-specific uptake and off-target cytotoxicity limit their clinical utility. To address these limitations, a cold atmospheric plasma-responsive delivery platform was developed in which the membrane activity of a peptide was transiently suppressed upon complexation with a DNA-based nanostructure. Upon localized plasma exposure, DNA masking was disrupted, restoring the biological functions of the peptides. Transmission electron microscopy revealed that the synthesized DNA nanoflower structures were approximately 150–250 nm in size. Structural and functional analyses confirmed that the system remained inert under physiological conditions and was rapidly activated by plasma treatment. Fluorescence recovery, cellular uptake assays, and cytotoxicity measurements demonstrated that the peptide activity could be precisely controlled in both monolayer and three-dimensional spheroid models. This externally activatable nanomaterial-based system enables the spatial and temporal regulation of peptide function without requiring biochemical triggers or permanent chemical modifications. This platform provides a modular strategy for the development of potential peptide therapeutics that require precise control of activation in complex biological environments.

## 1. Introduction

Cell-penetrating peptides have emerged as promising tools for the intracellular delivery of therapeutics because of their inherent ability to cross biological membranes without specific transporters [1,2]. Their small size, modularity, and synthetic accessibility render them attractive candidates for drug delivery, gene therapy, and imaging applications. However, their membrane-translocating capability is inherently nonspecific, which often leads to undesired uptake by non-target cells and dose-limiting cytotoxicity [3,4]. The lack of spatial and temporal control remains a critical barrier to clinical translation.

To address this challenge, stimulus-responsive strategies have been developed to temporarily suppress the activity of cell-penetrating peptides and reactivate them under specific conditions. One common approach involves the use of propeptides in which the active sequence is masked by a cleavable domain that is removed by enzymes in the target tissues [5]. Although enzyme-triggered activation offers biological specificity, its dependence on local enzyme expression limits its precision and consistency. Other strategies using pH- or redox-sensitive elements are associated with variability in endogenous conditions and lack of real-time external controls [6]. These limitations underscore the need for externally activatable peptide systems that remain inert during circulation and can be selectively triggered at the desired site.

Cold atmospheric plasma (CAP) is an emerging physical stimulus that generates reactive species near room temperature without direct contact with the target [7]. It has been used in cancer therapy, wound healing, and sterilization, and its use as a molecular switch for biomaterial activation is increasingly being recognized [8,9]. The short-lived nature of plasma-generated radicals ensures localized action with minimal systemic effects, making them compelling candidates for spatially confined activation of therapeutic agents.

**Scheme 1:**
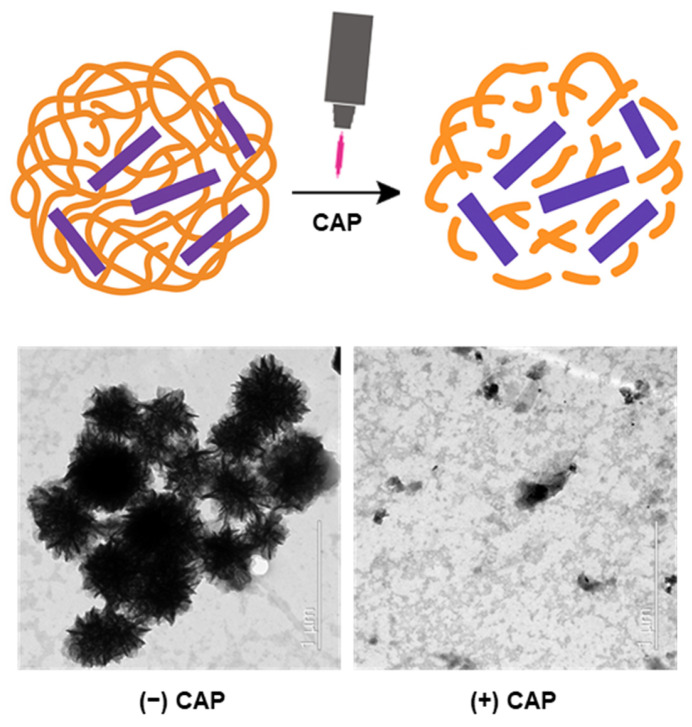
Schematic and TEM images illustrating the mechanism of cold atmospheric plasma (CAP)-responsive activation of a DNA-masked cell-penetrating peptide (PDNF). Upon CAP exposure, the DNA coating is disrupted, unmasking the peptide (free P) and restoring its bioactivity. TEM images show the disassembly of flower-like DNA structures and the dispersal of components after CAP treatment.

This study presents a CAP-responsive peptide delivery platform based on DNA masking. A membrane-active peptide is transiently inactivated by complexation with a DNA nanoflower (DNF) structure and is subsequently reactivated upon plasma exposure. Melittin, a representative cytotoxic peptide, was used as a model peptide in this study. The system was designed to remain inert under normal physiological conditions and become active only upon localized external stimulation. This approach enabled structural shielding, functional recovery, and selective cytotoxicity in both the monolayer and spheroid culture models. This platform provides a basis for externally guided peptide therapies that require precise control of the timing and location of activation (Scheme 1).

## 2. Results

### 2.1. Structural Disruption of DNF and Peptide-Encapsulating DNF by CAP

**Figure 1:**
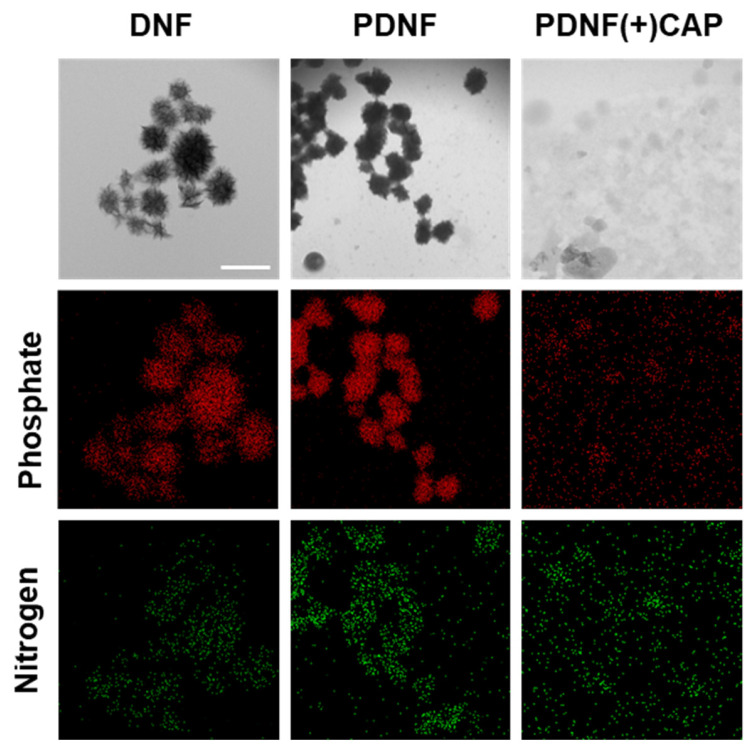
CAP-induced structural disruption and elemental redistribution of DNA-coated peptides. Morphological and elemental distribution analysis of flower-like DNA nanostructures before and after CAP treatment. TEM images (**top**) show compact assemblies in DNF and PDNF, which were disrupted upon CAP exposure. Elemental mapping of phosphorus (**middle**, red) and nitrogen (**bottom**, green) confirmed DNA (phosphorus-rich) coating around peptides and its removal by CAP. Scale bar, 500 nm.

To investigate the responsiveness of peptide-encapsulating DNF (PDNF) to external stimuli, structural changes were analyzed before and after CAP treatment. Transmission electron microscopy (TEM) revealed that both DNF and PDNF exhibited dense flower-like nanostructures prior to CAP exposure (Figure 1). The particle diameters measured in the TEM images were in the range of 150–250 nm, confirming their nanoscale characteristics. However, after 5 min of CAP treatment, the structures were disrupted and dispersed. Elemental mapping using energy-dispersive spectrometry (EDS) further demonstrated the presence of phosphorus (red) and nitrogen (green) signals corresponding to DNA and peptide components, respectively. The phosphorus signal significantly diminished after CAP treatment, supporting the effective removal of the DNA coating from PDNF.

### 2.2. Fluorescence Recovery Indicates Unmasking of the Quenched Peptide

**Figure 2:**
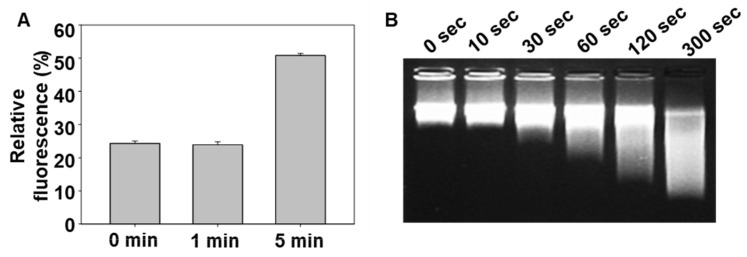
Kinetics of DNA uncoating from PDNF nanostructures triggered by CAP. (**A**) Relative fluorescence intensity of quenched dye released from PDNF upon CAP treatment over time. (**B**) Gel electrophoresis showing CAP-induced degradation or release of DNA from the nanostructures.

To confirm the DNA unmasking effect of CAP, a fluorescence quenching assay was performed using a co-encapsulated FAM-labeled peptide and a quencher-modified oligonucleotide within DNF. As shown in Figure 2A, the fluorescence intensity increased in a time-dependent manner after CAP exposure, indicating that the quencher was spatially separated from the fluorophore due to DNA disruption. Additionally, gel electrophoresis (Figure 2B) showed decreased DNA content in the CAP-treated group compared to that in the untreated control, further confirming DNA degradation or release upon CAP application.

### 2.3. CAP Restores the Cellular Uptake of DNA-Masked Peptide

**Figure 3:**
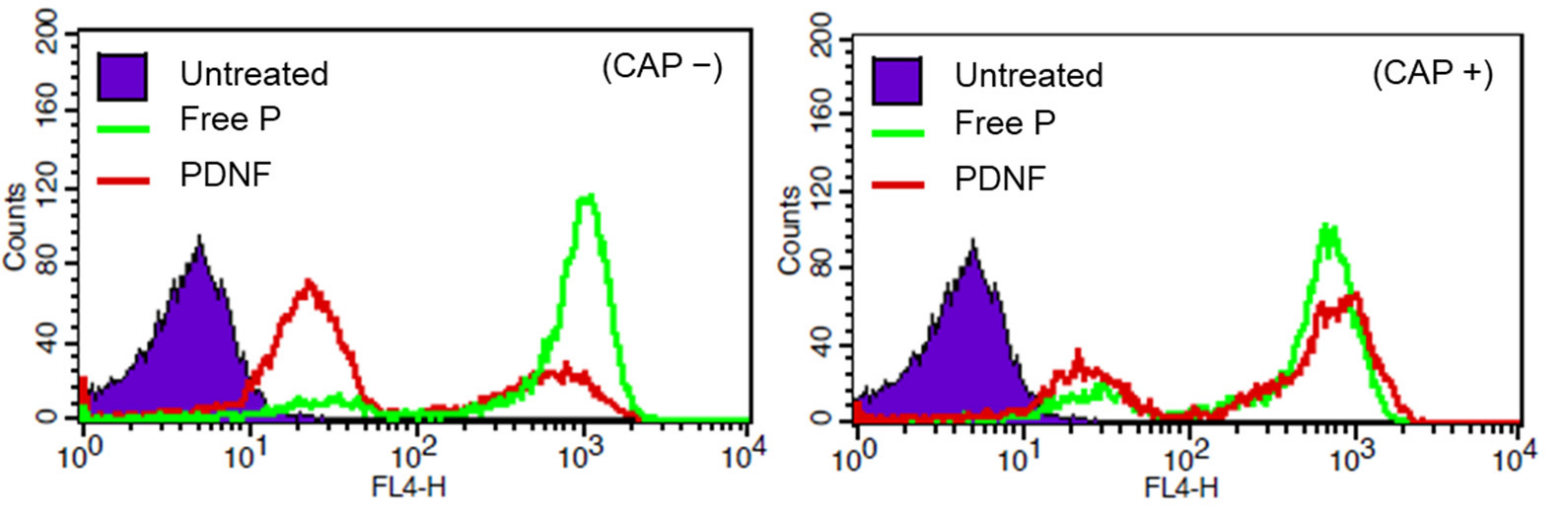
Cellular uptake of cell-penetrating peptides with or without CAP activation. Flow cytometric analysis of cellular uptake of free P, PDNF, and untreated control under CAP (−) and CAP (+) conditions.

Restoration of cell-penetrating activity following CAP-induced DNA removal was evaluated using cellular uptake analysis. Flow cytometric analysis (Figure 3) showed that under CAP (–) conditions, PDNF-treated cells exhibited lower fluorescence than those treated with free P, confirming that DNA masking reduces peptide internalization. In contrast, under CAP (+) conditions, PDNF-treated cells exhibited significantly higher fluorescence than that of the free P group, suggesting that peptide activity was restored by CAP-induced DNA uncoating.

### 2.4. Activated Peptides Penetrate 3D Spheroids upon CAP Exposure

**Figure 4:**
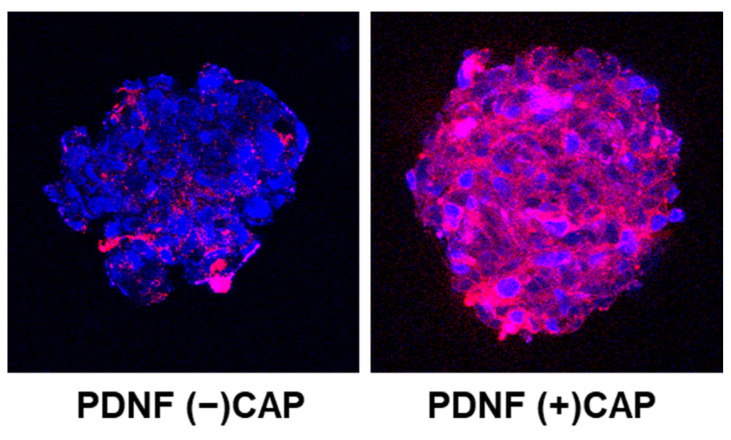
Intracellular distribution of fluorescently labeled peptide before and after CAP exposure. Confocal z-stack images of 3D cellular spheroids treated with Cy5.5-labeled peptide formulations. Blue: DAPI-stained nuclei; red: Cy5.5-labeled peptide.

To evaluate peptide penetration in a more physiologically relevant model, 3D tumor spheroids were used. As shown in Figure 4, confocal microscopy revealed limited Cy5.5 signal in the core of spheroids treated with PDNF under CAP (–) conditions, while CAP-treated PDNF showed a more homogeneous and deeper fluorescence distribution, similar to that of free P. This indicated that CAP effectively restored the cell-penetrating ability of PDNF, even in 3D tissue-like structures.

### 2.5. CAP Enhances Peptide-Induced Cytotoxicity

**Figure 5:**
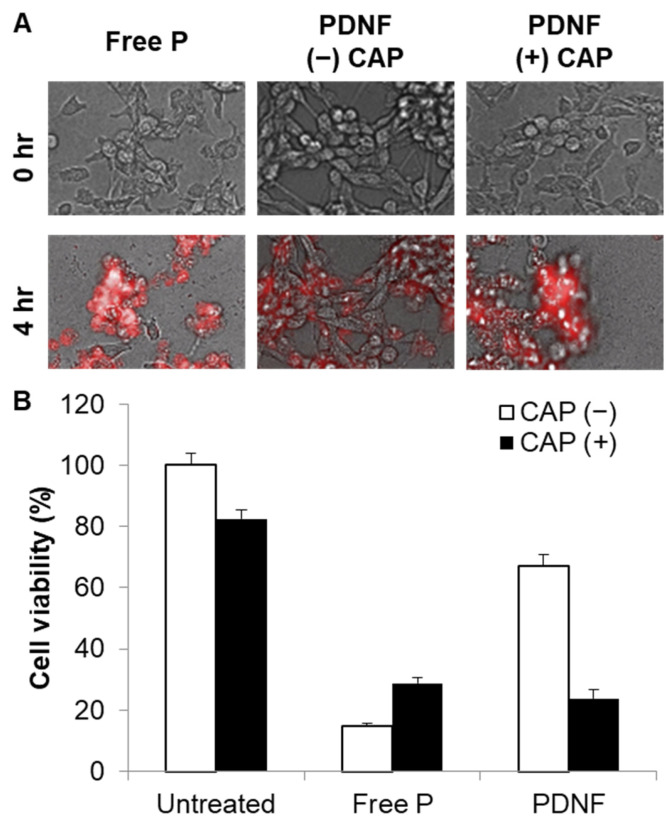
CAP-enhanced cytotoxicity of DNA-masked peptides in live cells. (**A**) Live-cell microscopy showing time-dependent morphological changes and intracellular Cy5.5 fluorescence of peptides 0 h and 4 h after treatment. (**B**) After 4 h, cell viability was determined using the MTT assay.

The biological effects of CAP-induced activation were further examined using live-cell imaging and 3-[4,5-dimethylthiazol-2-yl]-2,5 diphenyl tetrazolium bromide (MTT) assay. Live-cell fluorescence imaging over 4 h showed gradual accumulation of fluorescence and morphological changes in cells treated with CAP (+) PDNF (Figure 5A). Consistently, the MTT assay (Figure 5B) demonstrated that CAP (+) PDNF treatment significantly reduced cell viability compared with the CAP (–) or untreated groups, suggesting that peptide cytotoxicity was successfully reactivated following DNA unmasking by CAP.

## 3. Discussion

This study presents a stimulus-responsive peptide activation platform in which a cytotoxic cell-penetrating peptide is masked by DNF structures and subsequently reactivated by CAP exposure. This approach is based on electrostatic complexation between positively charged peptides and negatively charged DNA, resulting in temporary inhibition of peptide function. Upon application of CAP, the DNA shell is disrupted, leading to re-exposure of the peptide and recovery of its biological activity. This strategy offers a controllable delivery system that avoids permanent chemical modification of the peptide and utilizes an external physical stimulus to regulate peptide function, a concept aligned with emerging trends in externally controlled drug delivery platforms.

Morphological and elemental analyses confirmed CAP-triggered disassembly of the nanostructures. TEM imaging demonstrated the formation of compact flower-like DNA architectures in both DNF and PDNF formulations, which became disordered and dispersed following CAP exposure. The particle diameters measured using TEM ranged from approximately 150 to 250 nm, supporting the classification of these constructs as nanoscale assemblies. Elemental mapping using EDS revealed strong phosphorus and nitrogen signals corresponding to DNA and peptide components, respectively. A noticeable decrease in the phosphorus signal after CAP treatment supports the disruption of the DNA coating. This is consistent with previous observations that plasma-generated reactive species can damage nucleic acids via oxidative cleavage and strand breakage [10,11,12].

Functional unmasking of the peptide was verified on the basis of fluorescence recovery using a FAM-quencher-based assay. In this system, the close proximity of the FAM-labeled peptide and BHQ-oligo results in signal quenching, which is reversed upon disruption of the structure of the DNA complex. CAP exposure led to an increase in fluorescence, indicating separation between the fluorophore and quencher due to DNA disassembly. Electrophoresis confirmed a decrease in intact DNA bands after CAP treatment, consistent with DNA fragmentation. This dual confirmation confirms that CAP effectively triggers the structural and functional conversion of masked peptide carriers.

Restoration of the cell-penetrating function of the peptide was evaluated using cellular uptake analysis. Under CAP (–) conditions, the PDNF-treated cells showed reduced uptake compared with cells treated with the free peptide, demonstrating successful masking. In contrast, CAP-treated PDNF showed restored internalization levels, suggesting that DNA removal reinstated membrane-disrupting activity. This finding supports the concept of externally gated delivery systems that remain inactive until triggered, an approach distinct from enzyme [13,14] or pH-responsive platforms [15] and potentially more precise in its spatial application.

In a 3D tumor spheroid model, which better mimics in vivo tumor environments, CAP-activated PDNF displayed deeper and more uniform intratumoral penetration than non-activated controls. This suggests that DNA masking can inhibit diffusion in dense tissues, whereas external plasma exposure restores peptide function and enables entry into impermeable cellular architectures. Tumor spheroids provide a valuable platform for evaluating the penetration behavior of nanoparticle systems [16], and the results indicate that this system maintains responsiveness in more physiologically relevant contexts.

Finally, the cytotoxicity of the reactivated peptide was confirmed using live-cell imaging and MTT assays. In the absence of CAP, PDNF exhibited minimal toxicity, whereas CAP-activated formulations significantly reduced cell viability. This clearly demonstrates that masking effectively suppresses peptide function and that controlled activation via CAP leads to functional restoration. The concept of reversible noncovalent peptide masking for regulated cytotoxic delivery holds promise for therapeutic applications, including targeted cancer treatment and controlled immunomodulation [17]. The increased cell viability observed in the free P + CAP group may be attributed to minor effects of CAP-induced peptide decomposition. Although CAP alone is known to cause limited oxidative stress in biological molecules, the treatment parameters in our study were optimized to minimize decomposition-related effects in the absence of PDNF.

Although free peptides exhibit strong membrane activity, their application is limited by off-target toxicity and lack of spatial control. The PDNF system was designed to provide conditional activation, allowing the peptide to remain inert during systemic circulation and to be activated only at the desired site using CAP. This design aimed to reduce systemic toxicity and enhance therapeutic precision.

Although the in vitro findings clearly demonstrate the feasibility of CAP-induced peptide activation, several aspects remain to be addressed. The detailed mechanism via which plasma exposure selectively disrupts DNA structures while preserving the structural and functional integrity of peptides has not been completely elucidated. In addition, the potential non-enzymatic post-translational modifications of peptide residues under CAP-generated reactive species should be characterized [18]. Such modifications may influence biological function or stability, and their presence or absence should be confirmed using peptide-specific analytical techniques such as high-performance liquid chromatography or mass spectrometry to evaluate the integrity of the released peptides. In addition, considering that the effective penetration depth of CAP is limited to superficial tissue layers, delivery of CAP to deep-seated targets remains a technical challenge [19]. Future studies should explore minimally invasive delivery systems such as catheter-guided or endoscopic plasma devices, as well as plasma-activated liquid approaches to broaden clinical applicability [20].

## 4. Materials and Methods

### 4.1. Preparation of DNF and PDNF

Single-stranded DNA (DNF) was prepared via rolling circle amplification as described previously [17]. Briefly, 0.5 μM of phosphorylated linear single-stranded DNA (5′-ATC TGA CTA GTA TAT ACA AAA CTA ATG AGG CGT TGG AAG TGT AGT GGG GCG GTG CGC TCG GTC ATA GTA AT-3′, 45% GC content) was circularized using a primer and annealed with T4 DNA ligase (125 units/mL, Thermo Scientific, Waltham, MA, USA). The resulting circular template was incubated for 2 h with 1 μg/mL phi29 DNA polymerase (Thermo Scientific) and 2 mM dNTPs. After amplification, DNF was purified via centrifugation at 13,000× *g* for 10 min to remove unincorporated nucleotides. DNA concentration was measured using a Take3 microvolume plate (Agilent Technologies Inc., Santa Clara, CA, USA). To prepare DNA-coated peptide micelle complexes (PDNF), free P (H-Gly-Ile-Gly-Ala-Val-Leu-Lys-Val-Leu-Thr-Thr-Gly-Leu-Pro-Ala-Leu-Ile-Ser-Trp-Ile-Lys-Arg-Lys-Arg-Gln-Gln-NH_2_, Sigma-Aldrich, St. Louis, MO, USA) was mixed with DNF at a weight ratio of 10 in phosphate-buffered saline and incubated at room temperature for 10 min for electrostatic complexation.

### 4.2. CAP Treatment

CAP was used as an external trigger to remove the DNA shell from PDNF and activate the peptide. A CAP jet device (FLA Medic+; FLAMME Inc., Gyeonggi, Republic of Korea) operating with compressed air at a flow rate of 1 L/min was applied to the PDNF samples from a distance of 10 mm for 5 min. The samples were treated in a well plate or on coverslips, depending on the subsequent analyses.

### 4.3. TEM

The morphology of DNF and PDNF before and after CAP treatment was analyzed using TEM. A drop of each sample was deposited onto a carbon-coated copper grid and allowed to air-dry. Imaging was performed using a transmission electron microscope (JEM-2100F; JEOL, Tokyo, Japan), and element-distribution analysis was conducted using an EDS attached to the JEM-2100F.

### 4.4. Fluorescence Assay for DNA Unmasking

A fluorescence quenching assay was used to monitor CAP-induced unmasking of the peptide. The FAM-labeled peptide and a short oligo modified with Black Hole Quencher 1 were co-encapsulated within DNF during the formation of PDNF complexes. The samples were treated with CAP and incubated for 0, 1, and 5 min. Fluorescence intensity was measured using a microplate reader (BioTek, Winooski, VT, USA) at excitation and emission wavelengths of 488 nm and 520 nm, respectively. The fluorescence signal was normalized to that of the untreated controls.

### 4.5. Gel Electrophoresis

Gel electrophoresis was performed to confirm CAP-induced DNA release. CAP-treated samples were mixed with a pink staining dye (GenDEPOT, Barker, TX, USA) and loaded onto a 3% (*w*/*v*) agarose gel. Electrophoresis was performed at 120 V for 15 min, and the gel was imaged using a ChemiDoc system (ProteinSimple, Santa Clara, CA, USA).

### 4.6. Cellular Uptake

To evaluate cellular uptake, 4T1 cells were seeded in 12-well plates and treated with free P, PDNF, or CAP-treated PDNF for 30 min. After treatment, the cells were washed, trypsinized, and resuspended in phosphate-buffered saline for analysis using a flow cytometer (NovoCyte Advanteon Flow Cytometer; Agilent Technologies). To evaluate the penetration of the peptide formulations into 3D cellular structures, spheroids were generated using 4T1 cells, as described previously [21]. Spheroids were treated with Cy5.5-labeled free P, PDNF, or CAP-treated PDNF for 30 min. After treatment, the spheroids were stained with DAPI and observed using confocal fluorescence microscopy (LSM 710; Carl Zeiss, Oberkohen, Germany).

### 4.7. Cytotoxic Effect

Cytotoxicity was assessed using live-cell microscopy and MTT assay. For live-cell microscopy, 4T1 cells were seeded into 48-well plates and treated with free P, PDNF, or CAP-treated PDNF. During incubation for 4 h in an environmental chamber of a fluorescence microscope maintained at 37 °C, 5% CO_2_, and 100% humidity, images were obtained at 30 min intervals (Lionheart FX Automated microscope). For the cell viability assay, 4T1 cells were seeded in 96-well plates and treated with free P, PDNF, or CAP-treated PDNF. Following a 4 h incubation, the culture medium was removed, and 100 μL of dimethyl sulfoxide (GenDEPOT) solution was added. The plates were incubated at 37 °C until the formazan dissolved completely, and cell viability or death was assessed by measuring the absorbance of dissolved formazan at 570 nm using a multi-mode reader. Cell viability for each group was calculated as a percentage relative to that of the control cells.

### 4.8. Statistical Analysis

Statistical data were analyzed using Student’s *t*-test or ANOVA, followed by the post hoc Student–Newman–Keuls test. SigmaPlot (Version 14.5, Systat Software Inc., San Jose, CA, USA) was used for all data analyses, and *p*-values less than 0.05 were considered significant.

## 5. Conclusions

This study presents a novel platform for the external control of peptide activity using a CAP-responsive DNA-masking strategy. By using DNF structures to transiently inhibit the function of cell-penetrating peptides, peptide activity was effectively suppressed under normal conditions and reactivated in response to CAP exposure. Structural characterization confirmed CAP-triggered disassembly of the DNA shell, and functional analyses demonstrated the recovery of cellular uptake and cytotoxicity upon unmasking. The system showed high versatility across both 2D and 3D cell culture models, indicating its potential for complex biological applications. In particular, the ability to regulate cell-penetrating peptide penetration into tumor spheroids underscores their relevance in targeted therapeutic strategies. This approach allows for precise external and non-contact activation, offering spatiotemporal control without modifying the peptide sequence. Taken together, these findings demonstrate the feasibility of CAP-guided peptide activation using reversible DNA masking as a generalizable strategy for controlled drug delivery. This platform can be extended to a broad range of therapeutic peptides and represents a promising direction for designing stimulus-responsive biomolecular systems for precision medicine.

## Data Availability

Dataset available upon request from the authors.
